# What is an ideal mHealth for post-discharge home care of CHD: perspectives from primary caregivers, healthcare professionals, and project technical consultants: a qualitative research study

**DOI:** 10.3389/fcvm.2026.1948803

**Published:** 2026-10-20

**Authors:** Ruijie YangLan, Fang Ma, Qinglan Li, Wantong Yao, Shu Peng

**Affiliations:** 1 School of Nursing, Kunming Medical University, Kunming, China; 2 First Affiliated Hospital of Kunming Medical University, Kunming, China

**Keywords:** congenital heart disease, mHealth, post-discharge home care, reflexive thematic analysis, stakeholder-aligned design

## Abstract

**Background:**

Congenital heart disease (CHD) remains a leading global birth defect with high prevalence, yet advances in diagnosis and treatment have raised survival rates to 90%–95%. This epidemiological shift transforms CHD into a chronic condition demanding long-term post-discharge home management. Although mobile health (mHealth) holds promise for supporting such care, current tools suffer from low adherence, insufficient age-appropriateness, and a lack of emotional and social support, and most evaluations neglect the integrated perspectives of primary caregivers, clinicians, and technical developers.

**Objective:**

This study aims to explore and synthesize the needs for and perspectives regarding an idealized mHealth system for post-discharge CHD home care from the perspectives of primary caregivers (PC), healthcare professionals (HP), and technical consultants (TC).

**Methods:**

A qualitative descriptive design was employed, using semi-structured interviews with seven PC, six HP, and three TC. Data were analyzed using Braun and Clarke's Reflexive Thematic Analysis (RTA) framework.

**Results:**

Six overarching themes were identified: (1) tailored to individual, (2) easy to use, (3) media in convergence, (4) at the ready, (5) one clear voice and (6) emotional support on demand.

**Conclusions:**

These findings provide a stakeholder-informed framework to guide future development of user-centered, clinically relevant, and technically robust mHealth interventions.

## Introduction

Congenital Heart Disease (CHD), also referred to as “congenital heart defect”, is a category of congenital malformations characterized by abnormalities in cardiac morphology and vascular function. These anomalies arise from disturbances in the formation and development of the heart and great vessels during fetal embryogenesis, or from the failure of physiological channels that are expected to close spontaneously after birth to undergo normal closure (1). According to data from the Global Burden of Disease Study 2019, the global number of individuals living with CHD had reached 13 million, with a mean birth prevalence of 8.2 per 1,000 live births (2). As one of the countries with a high burden of CHD worldwide, China reports a birth prevalence of 8.98 per 1,000 live births, with an estimated 2 to 4 million people living with CHD and an annual increment of 150,000 to 200,000 new cases (2–4). CHD represents a significant global health burden, ranking as the leading cause of mortality among all birth defects (1). As a result of advances in diagnosis and treatment, the survival rate for children with CHD into adulthood has now reached 90%–95% (5–7). Consequently, CHD has transitioned from an acutely life-threatening condition to a chronic disease requiring long-term management, which highlights the crucial, yet often under-supported, phase of post-discharge home care. Studies indicate that late mortality in CHD survivors is frequently linked to non-cardiac causes, such as pneumonia, underscoring the need for meticulous home-based management to ensure the continuity of care and improved long-term outcomes (8).

Mobile health (mHealth) is defined by the World Health Organization as medical and public health practices supported by mobile devices, such as smartphones, tablets, and wearable sensors, as well as wireless communication technologies (9). mHealth has emerged as a promising tool to facilitate chronic disease management, offering the potential for enhanced patient engagement, remote monitoring, and continuous support. Current evidence from randomized controlled trials and guideline recommendations has confirmed the effectiveness of mHealth in perioperative rehabilitation and the remote follow-up stage in cardiovascular disease management, such as hypertension and atrial fibrillation, demonstrating positive effects in behavioral change, adherence, and certain clinical outcomes (10, 11). Recently, mHealth has been developed and applied in post-discharge home care of CHD, showing obvious improvements in patients' outcomes. A scoping review by Li et al. suggests that existing mHealth supporting CHD family care performs well in information access and care coordination (12). A previous study demonstrated (13) that an mHealth-based cardiac rehabilitation program effectively improved cardiorespiratory fitness and physical activity in youth with CHD. Schöneburg et al. utilized mHealth approaches integrating gamification, demonstrating the feasibility and user acceptance of the app-based home exercise program for children and adolescents with CHD (14).

Despite the growing interest in mHealth for CHD, some weaknesses were reported by researchers, including low adherence to mHealth, not being sufficiently age-appropriate, deficiencies in motivational incentives, lack of social support and emotional connection (12). In addition, present studies predominantly evaluate efficacy only from a patient-centric perspective, without establishing a comprehensive understanding of what constitutes an ideal mHealth tool. The perspectives of multiple crucial stakeholders—namely, the primary caregivers (PC) who manage daily care, healthcare professionals (HP) who provide clinical oversight, and technical consultants (TC) who design and build these systems—are rarely integrated. This study, therefore, aims to explore and synthesize the needs for and perspectives of an ideal mHealth for post-discharge CHD home care from these three integral perspectives. By identifying a shared vision for an ideal mHealth system, we seek to move beyond a narrow focus on clinical efficacy to a more holistic understanding of utility, acceptability, and feasibility. Our findings are intended to inform the future development of user-centered, clinically relevant, and technically robust mHealth interventions that can genuinely support the growing population of children with CHD and their families during their post -discharge home care.

## Methods

### Study design

This study adopted a qualitative descriptive design, which is widely used in healthcare research to provide direct, rich descriptions of phenomena and to inform practice. This design aligns with our aim to explore and describe the needs for and perspectives of multiple stakeholders regarding an ideal mHealth for post-discharge CHD home care. We employed Braun and Clarke's Reflexive Thematic Analysis (RTA) framework to guide data collection, analysis, and reporting. One-on-one, semi-structured, in-depth interviews were conducted with PC, HP, and TC. Each participant group used a separate semi-structured interview guide containing open-ended core questions and flexible probes, which allowed participants to elaborate on their experiences and raise issues not anticipated by the researchers.

This study was reported in accordance to the Consolidated Criteria for Reporting Qualitative Research (COREQ) checklist (15), which was used to enhance the transparency and quality of the final reporting.

### Study setting, participants, and sampling method

This study was conducted in the cardiology and cardiovascular surgery wards of a tertiary hospital in southwestern China. Participants included PC of children with CHD, HP from the same departments, and TC from our collaborative team. Purposive sampling was used. Inclusion and exclusion criteria are presented in Table 1.

**Table 1:** Consolidated criteria for reporting qualitative studies (COREQ): 32-item checklist.

Research participants	Inclusion	Exclusion
Primary Caregiver	(1) The child was diagnosed with CHD and had undergone interventional catheterization or surgery;	(1) Unable to independently operate a smartphone or had no experience with mobile health;
	(2) The caregiver was a first-degree relative (parent or sibling >12 years old) and could clearly describe their experiences, perspectives, and needs in Mandarin;	(2) Limited communication ability, such as only understanding the local dialect, making them unable to cooperate in semi-structured interviews.
	(3) They agreed to participate.	
Healthcare Professional	(1) Held a valid medical practitioner license or nursing license and currently worked in cardiovascular medicine or surgery;	(1) Had taken leave due to illness, maternity, or other reasons in the past 3 months;
	(2) Had at least 2 years of front-line clinical experience in the cardiovascular field;	(2) Simultaneously participated in other related medical or research projects.
	(3) Had regularly used mHealth systems or tools from their institution or other relevant sources in the past 6 months.	
Technical Consultant	(1) Currently or within the past 2 years, had worked in technology development related to mHealth;	(1) Had projects under confidentiality or non-compete agreements;
	(2) Had experienced in selecting technical solutions or solving patient problems based on real medical scenarios;	(2) Their team had experienced a major online production incident within the past 3 months and was still under liability determination.
	(3) Voluntarily participated and could clearly recall and reflect on requirement conflicts, technical constraints, and user experience iterations during development.	

### Data collection

For each participant group (PC, HP, and TC), a separate preliminary semi-structured interview guide was developed based on a literature review and revised after pilot interviews. Prior to the formal interviews, the research team conducted three pilot interviews, with the sample comprising one PC, one HP, and one TC. All pilot participants met the same inclusion criteria. Their data were not included in the final analysis and were used solely to assess the appropriateness and readability of the interview guides. Based on the pilot feedback, the guides were revised as follows: some terms were replaced with more accessible language, and a brief explanation of mHealth was provided to participants before the interview. Given that caregivers spontaneously raised emotional concerns related to caregiving, a probe question addressing emotional experiences was added to the final guides. Finally, overlapping questions were consolidated to reduce the estimated interview duration from 45 to 55 min to 20–45 min, so as to minimize the participation burden on caregivers. The final versions of the interview guides are provided as Supplementary Appendix 1.

The interview guides mainly included the following: (1) needs for and perspectives regarding an ideal mHealth for post-discharge CHD home care and (2) expectations about the development of mHealth. The principal investigator (YL) contacted potential participants to introduce the study procedures. An informed consent form was distributed to all potential participants, providing a comprehensive explanation of the research objectives, as well as the rights to confidentiality and voluntary participation. Participants were also informed that all research data would be anonymised. Prospective participants were given a 24-hour consideration period to decide whether to enrol in the study. Written informed consent was obtained from all participants on site prior to each interview, thereby securing formal authorisation for their involvement in the study. All interviews took place in a private room in the hospital. Interviews were conducted in Chinese, lasted 20–45 min, were audio-recorded with consent, and transcribed verbatim within 24 h. The researcher maintained neutrality and empathy throughout.

### Data analysis

We used RTA as developed by Braun and Clarke, located within a Big Q qualitative orientation in this study (16, 17). This approach reflects the values and sensibilities of a qualitative paradigm and directly attends to the researcher's role and interpretive process throughout the analysis (18). This updated approach is intended, in part, to distinguish itself from earlier styles of thematic analysis—such as codebook, coding reliability and framework analysis—which adopt a more structured and often combined inductive-deductive logic (18). Within RTA, themes are conceptualised as patterns of shared meaning underpinned by a central organising concept, rather than as domain summaries or topic-based categories (16). Coding quality in RTA stems not from consensus between coders, but from depth of engagement with the data and situated reflexive interpretation. This process-based, organic, and evolving orientation to coding makes saturation an ill-fitting notion for reflexive TA. Accordingly, we agree that data saturation should be avoided in this approach, and that data adequacy offers a more suitable framework for justifying sample size decisions (18, 19). Consistent with this orientation, we did not seek coding consensus, inter-coder reliability, or completeness.

We located the analysis within a critical realist ontology and a contextualist epistemology. This positioning means that participants' accounts were treated as meaningful representations of their experiences and social contexts, while analysis remained interpretative, situated, and reflexive rather than aiming to discover an objective truth waiting in the data (16). This epistemological and ontological stance informed our analytic decisions and our reporting of themes as interpretative patterns of shared meaning.

Qualitative data were managed in NVivo 12. All transcripts were anonymized before analysis. RTA followed Braun and Clarke's six-step framework (17, 20) as the primary analytical approach. Two researchers (YL and Ma) participated in this process: YL conducted the full analysis, while Ma served as a senior qualitative research scholar and reflexive collaborator, providing critical friendship rather than acting as a second coder or consistency verifier. Both researchers repeatedly read transcripts to gain data familiarity, then performed line-by-line coding to generate initial codes. Through an iterative process of comparison, interpretation, and integration, codes were clustered into candidate themes based on shared meaning and a central organising concept. A recursive review examined the coherence and interpretive fit between candidate themes, coded extracts, and the full dataset. Finally, an analytic report was written, cross-reviewed, and revised by all researchers to enhance reflexive rigour. All analysis was performed using original Chinese transcripts to preserve nuance. Reflexive practice is a cornerstone of RTA, which treats researcher subjectivity as a resource rather than a problem to be controlled. In keeping with this orientation, we offer the following reflection on our positionality and how it may have shaped the analytic process. The analysis was primarily conducted by YL, a master's degree candidate in nursing who had received systematic training in qualitative research. He is a partial insider to the experiences of the participants. While he has not personally used the mHealth intervention for post-discharge home care of children with CHD, he was involved in its development alongside TC. This positioned him to bring both experiential familiarity and a critical awareness of the healthcare context to the analysis, while also requiring ongoing reflexivity about how his prior experiences might predispose him toward certain interpretations. The analysis was further discussed and refined through regular meetings with the second author, whose previous research on the healthcare needs of patients with chronic illness provided a theoretical lens through which the data were approached. These collaborative reflexive discussions helped to ensure that the interpretations were not unduly shaped by any single viewpoint, but rather developed through a dialogical engagement with the data across different subjectivities.

In the early stage of the study, the research team experimented with large language models (LLMs) as a brainstorming tool to assist coding, though this exploratory approach was not incorporated into the final analytical framework. Braun and Clarke argued that deploying LLMs as a “second coder”, consistency verification tool, or a means to ensure research integrity is incompatible with RTA (21). Following methodological reflection, AI-generated coding was not incorporated into the final analysis, and the reported analysis was subsequently conducted entirely by the research team.

### Translation for publication

After analysis was complete, original Chinese materials supporting the findings were translated into English by two independent bilingual translators with medical backgrounds using a forward-and-back procedure, with cross-checking to preserve original meaning. This step was undertaken to prepare the data for presentation in an English-language manuscript.

### Ethical considerations

This study was approved by the Ethics Committee of the First Affiliated Hospital of Kunming Medical University (2025L393) and adhered to the Declaration of Helsinki. This study has been registered in a clinical trial registry, NCT07385209. All participants provided written informed consent after full disclosure of study details, including the right to withdraw without consequence. All materials were stored on encrypted drives accessible only to the research team, and all materials will be securely destroyed within two years post-project completion.

## Results

The final sample consisted of seven PC of children with CHD, six HP, and three TC. Among the PC, there were six mothers and one father, with ages ranging from 22 to 38 years. Their educational attainment varied from primary school to bachelor's degree, and their places of residence covered both rural and urban areas. The HP included four female nurses and two male physicians, aged 22 to 47 years. The majority held a bachelor's degree (*n* = 5), with one holding a master's degree. Their professional titles ranged from junior to intermediate levels, including junior nurse, supervisor nurse, and attending physician. Regarding years of specialty practice, two had ≤3 years, two had 4–7 years, and two had ≥10 years. Their roles involved cardiac surgical nursing and cardiac surgery. The TC were all male, aged 25 to 26 years, and all held master's degrees in software engineering. They had previously undertaken roles such as back-end development engineer or requirements analyst in mobile health projects and possessed relevant practical experience in the field. Detailed demographic characteristics of the participants are presented in Appendix 2. A total of six overarching themes were generated jointly through analyses conducted by the researchers.

### Theme 1: tailored to individual

Participants expressed that the mobile health needed to provide information and recommendations that were accurately applicable to an individual patient's condition, avoiding generic or formulaic content that may cause deviation. They highlighted the function of mHealth to capture patient-specific differences, including specific illness details, treatment methods, comorbidities, allergies, family environment, genetic background, and regional culture, and to offer personalized solutions accordingly. It was noted by PC that existing mHealth apps were too generic and lacked patient specificity. Although the information itself may be factually correct, its lack of specificity to her child renders it practically useless. They considered this one-size-fits-all development model to be of limited practical value.

“I have used apps before to find out when surgery would be best, how it would affect my child, and what the harm might be. But I found it inconvenient and not very useful. Every child's condition is different.”—PC 1

HP also noted that individual patients have undergone different treatments and procedures, which highlights the need for personalized health guidance and education.

“Open-chest surgery and interventional surgery have different post-operative exercise restrictions. But current models cannot provide personalized exercise prescriptions; They mostly give general content. While the general content is not wrong, it is certainly not the best.”—HP 3

### Theme 2: easy to use

Participants stated that they generally disliked intrusive designs such as complicated registration processes and frequent push notifications; instead, a simple interface and low learning cost were better. They emphasized that the tool should directly serve problem-solving rather than flashy features.

HP stated that mHealth apps should make their work easier, not increase their workload. Excessive complexity would only lead them to abandon adoption.

“The app has patients’ medical records and related health education content, and it integrated multiple functions into one platform. We could check laboratory reports and deliver patient education resources easily and quickly without leaving the app, which made our routine follow-up work much more convenient. ”—HP 3

TC also noted that, although complex development might be chosen by some technicians to showcase their professional skills, the ultimate objective should be simplicity and user-friendliness. Given that the app was designed to be user-oriented, its primary purpose was to help users resolve their issues with minimal effort.

“Some of my peers like to create complex health data charts, fancy points systems, and pop-ups every day asking users to fill in information. But who is the user? She is a mother who just went through her child's surgery, exhausted, unable to sleep well at night. She opens the app wanting only to solve her child's problem as quickly as possible.”—TC1.

### Theme 3: media in convergence

Notably, our study found that TC emphasized algorithm optimization during the development of mHealth, which might, either for communication between PC and HP or for guiding PC, result in misunderstanding and boredom.

Whereas diverse kinds of media like video, pictures, and stories can facilitate communication and guide PC to better grasp the skills for caring.

HP stated that when parents consulted them about their children's signs and symptoms, verbal descriptions alone were often hard to understand. Videos or pictures proved to be a useful medium for helping HP grasp the patient's condition, thereby facilitating appropriate clinical decision-making and treatment.

“Through video, we can see the child's actual condition. For example, sometimes a parent tells me the child is breathing poorly. By looking at the video, we might see that the child is actually fine. Alternatively a family member calls saying the wound is not healing well or there is a stitch. In such cases, we just need to take a look. If there is no major problem, the patient does not need to travel to the hospital.”—HP 5

PC also reported videos facilitated better comprehension of medical information, while concise text could provide key information—both of which collectively contributed to keeping patients adequately informed.

“I hope that when I search, there will be images, text, and videos. Short videos can make things less tedious, or make complex procedures easier to understand. If I want to extract information quickly, some videos are too long, and precise text is the priority, which might take me only one or two minutes to find the information”-PC 3

TC echoed this view, noting that some people preferred video because they had difficulty understanding text-based information, and that multimedia was an effective way to meet the diverse needs of different users.

“Some PC users have low education levels. If you give them a 3,000-word health article, they will get a headache. But if you give them a 40-second video, they might be willing to watch it several times. Multimodal development in software is also meant to meet the needs of different people and retain more users.”—TC3.

### Theme 4: at the ready

PC and TC mentioned that real-time responsiveness constituted a core attribute of desirable mHealth tools, and user retention hinged on whether users' problems could be resolved promptly upon emergence.

PC stressed the importance of rapid response and real-time interaction of mHealth apps. They expressed that after their children with CHD were discharged, they often found themselves in home environments with limited access to healthcare resources, far from the hospital, where acute concerns arising in daily care could not be promptly addressed through in-person visits. In this context, the real-time responsiveness of mHealth was a core compensatory mechanism that bridges the spatial and temporal gaps in healthcare accessibility.

“We do not live in Kunming. If my child has a problem, we cannot go to the hospital immediately. Although we have a WeChat group, the doctors are too busy. Some questions go unanswered, so it feels not very useful. Unlike AI, where you ask and get an immediate reply, that would be very convenient for me.”—PC 2

### Theme 5: one clear voice

Participants pointed out that they might receive inconsistent information regarding the same issue, leading to confusion in family decision-making. They stressed that information from mHealth should align with the clinical judgment of HP, avoiding patient-provider conflicts caused by generic, templated advice. In addition, information from different mHealth must be the same, following up-to-date scientific evidence.

PC expressed their concern about the conflicting information from different apps, which amplified their uncertainty and made them confused.

“My husband and I have both used health-related apps to ask questions about our child. But the answers from the two apps seem different.”—PC 2

TC also raised concerns regarding information consistency, elaborating that in the development of mHealth applications, the accuracy of health information and data consistency ought to be prioritized over algorithmic optimization.

“When designing software, engineers might connect to several knowledge bases, or the ranking logic of the search engine may have randomness. Some engineers do not see this as a bug; they call it “algorithm optimization.” But for users, this is a principled error. Medical problems are not about “customers who liked this also liked that.” What users need is a certain and authoritative answer, not a probability.”—TC1

“If we do not pay attention to data consistency early on, all kinds of strange and bizarre bugs will emerge later. That not only reduces user experience but also forces us to waste time fixing these avoidable problems.”—TC2.

### Theme 6: emotional support on demand

Participants pointed out that mHealth should be able to recognize and respond to the loneliness, anxiety, and psychological needs of patients and their families during the course of illness, thereby filling the gap left by HP, who often had limited time to provide emotional support. Emotional support can be reflected in features such as online experience-sharing platforms, which may help users gain social support, reduce stigma, and realize that they are not alone, thus alleviating the anxiety caused by the disease.

HP acknowledged that, despite their recognition of the importance of providing emotional support and comfort to patients and families, such practices were frequently impeded by time limitations. An mHealth application equipped with this functionality would therefore be a valuable support tool.

“PC may need comfort when facing the disease, often from the doctors. But we do not have enough time to comfort them. If there is a platform where they could vent and talk about their feelings, I think that would be very meaningful.”—HP 1

“Some people may not be willing to talk about the disease, considering it as their privacy. An online platform with an anonymous feature is a good approach. There, without worry, they can share with others and ask questions freely.”—HP 2

PC also expressed their longing for emotional support through mHealth.

“ Hope there is a platform online where PC can communicate with each other, which is definitely emotional support for us.”—PC 3

## Discussion

Through in-depth interviews with PC, HP, and TC about their needs for and perspectives regarding an ideal mHealth for post-discharge home care of CHD, this study identified six parallel themes. The parallel generating of these themes indicates that optimal mHealth interventions for post-discharge CHD care are not determined by a single factor, but rather shaped by the simultaneous interplay of multiple dimensions. Recognizing this constellation of themes is essential for designing comprehensive mHealth strategies that address the broader contextual landscape rather than isolated issues. Based on the needs and perspectives identified from different stakeholder groups, this study found that from the perspectives of the PC, HP, and TC, an ideal mHeath for post-discharge home care of CHD includes features such as tailored to individual, easy to use, media in convergence, at the ready, one clear voice, and emotional support on demand.

Tailored to individuals is considered by the three stakeholder groups as one basic functional requirement. Users' demand for tailored information reflects a need to reject generic content and receive information tailored to individual differences (22). Existing research has shown that perceived personalization can influence usage intention through attitudes and further relate to health behavior change (22). It is also evidenced by Li that personalization may represent an important mechanism supporting engagement and sustained use in home-based exercise programs for children and adolescents with CHD (13). Furthermore, our study suggests that “tailored to individual” not only addresses matching individual preferences, but also stresses condition-specific adaptation, Such as disease severity, surgical type, medication regimens, comorbidities, family environmental factors, and health literacy levels of different caregivers.

In addition, ease of use is another functional requirement for the participants. According to the Technology Acceptance Model (TAM) theory (23), users' actual adoption or rejection of mHealth can be affected by perceived ease of use. It is also evidenced by Jin et al. that whether mHealth is easy to use can facilitate the use of mHealth, and it is recommended that the development of mHealth should focus on simplifying operational procedures (24). An ideal mHealth should take the simultaneous satisfaction of being tailored to individual and ease to use as its basic prerequisite, and these two together constitute the core foundation for user acceptance and continued use.

In addition to the fulfillment of basic functional requirements, users have also explicitly articulated their requirements for media in convergence. According to the Extended UTAUT2 Model, hedonic motivation, which refers to fun, pleasure, and enjoyment with the use of technology, is widely considered an important predictor of mHealth acceptance (25). The value of multimodal content such as videos and images lies not primarily in being “interesting,” but also in lowering the cognitive threshold for health information, acting as a compensatory pathway for low-health-literacy populations to acquire essential care knowledge. Furthermore, in the context of telenursing, nurses can develop educational resources such as e- brochures and easy-to-understand videos (26). These materials can be integrated into mHealth to accommodate patients' learning levels and cultural specificity.

Meanwhile, mHealth's “at the ready” feature is not merely a functional preference, but a core mechanism that alleviates information gaps and decision-related anxiety. These materials can be integrated into mHealth, which can suit PCs' learning levels or cultural specificity, and mHealth that is rich in presentation forms becomes key digital infrastructure for knowledge transfer in remote home care. For PC of children with CHD during the post-discharge home care phase, the “at the ready” feature of mHealth is not merely a functional preference, but rather constitutes a core supportive mechanism that alleviates information gaps and decision-related anxiety during the home-based recovery period. Furthermore, traditional Chinese culture has long advocated the principle that “diseases should be treated in their early stages,” a notion that fosters high expectations among PC and patients regarding the efficiency of medical services. Chen et al. (27). Similarly demonstrated that excessively long waiting times or delays in care processes are prone to trigger PC and patients' dissatisfaction. A national survey on electronic consultations in Norway further revealed that 68.7% of users expected a reply within 24 h (28). However, despite patients' general preference for “the faster, the better” in terms of response speed, the current healthcare system has not established “real-time response” as a rigid standard for all online services (29). Response delays not only diminish patient satisfaction but may also exacerbate their anxiety. Akiogbe et al. (30) found that when patients are directly presented with test results bearing “red abnormal flags” in the absence of explanatory materials, they are prone to overinterpretation and subsequent panic. Hence, the design of mHealth interactivity should be based on multimodality and timeliness. These two elements are complementary and constitute a critical pathway for improving satisfaction, alleviating anxiety, and promoting health equity. Future platforms should continue to make sustained efforts in both accelerating speed and achieving deeper integration with interactive content.

In the study, users have further raised demands for the authority and consistency of mHealth content. Conflicting information in different channels might trigger distrust toward mHealth. Some studies have evidenced that trust is a predictor (25, 31, 32) of the adoption of mHealth, highlighting the key role of information consistency. Moreover, according to cultural dimension theory, Chinese individuals have a medium level of uncertainty avoidance tendency. When parents face the uncertainty of disease trajectory, they might seek diverse knowledge and information to reduce discomfort brought by uncertainty (33). Liu et al. pointed out that to enhance the reliability of their judgments, Chinese patients would cross-verify information by conducting cross-platform or multiple searches (34). Hence, the evidence base emphasizes the importance of the provision of consistent information in mHealth.

Our study reveals that caregivers' needs for mHealth manifest as an essential demand for emotional connection and psychological support in the digital space. Notably, our study found that TC emphasized algorithm optimization during the development of mHealth, which might be related to their training experiences in technology-driven design. This is also supported by other research indicating that differences exist between developers and clinicians in AI development—including opposing goals (model interpretability vs. clinical plausibility), different sources of truth (data vs. patient), and the tension between exploring new knowledge and exploiting existing knowledge. Furthermore, while developers treat training data as the source of truth, clinicians prioritize patient-specific context, which can lead to contradictory interpretations (developers treat training data as the source of truth while clinicians prioritize patient-specific context). Evidence indicates that mHealth's lack of empathy, insight into users' emotions, and adaptive responses exacerbates caregivers' uncertainty, anxiety, and decisional loneliness (33, 35). In this study, caregivers sought an anonymous platform to share experiences and receive peer support, echoing Fu et al.'s emphasis on continuous care and feeling “seen and remembered” (36). Therefore, we advocate for purpose-driven, patient-centered solutions with embedded emotional support rather than a technology-first approach. An interdisciplinary network involving experts from academic, industrial, clinical, ethical, legal, and regulatory domains is highly recommended (37).

Compared with the needs for and perspectives regarding stakeholders (PC, HP, and TC) regarding mHealth for CHD reported by our study, those from current studies on patients' perspectives show both similarities and differences. Patients also indicated that usability, credible content, and customization to patient needs are key elements of an ideal mHealth app. Notably, enjoyment were features mentioned only by patients, which may be related to their age-appropriate characteristics—children tend to enjoy games and seek enjoymentc (14, 38). Combined with the needs and perspectives of patients, our findings contribute to the improvement of mHealth for CHD.

### Strengths and limitations

This study has several notable strengths. First, it incorporates perspectives from three categories of stakeholders. Compared with studies adopting a single-perspective approach, it enables a more comprehensive and cross-validated understanding of mHealth-related needs. In addition, the identified thematic categories of stakeholder demands provide evidence and actionable design recommendations for the development of mHealth interventions for patients after CHD surgery.

We acknowledge several limitations. First, the TC subgroup included only three participants, a constraint driven by the objective scarcity of professionals with both deep mHealth development experience and medical domain familiarity. Our aim was information richness rather than statistical representativeness; While this approach is defensible for highly specialised populations, we concede that three consultants may not fully capture the diversity of professional perspectives, limiting transferability. Nonetheless, core themes from this subgroup—such as information consistency, ease of use, and media convergence—were corroborated by themes independently derived from PC and HP. The study was conducted at a single tertiary hospital, which may restrict generalisability to other settings with differing resources or regional cultures.

## Data Availability

The original contributions presented in the study are included in the article/Supplementary Material, further inquiries can be directed to the corresponding author/s.
