# Supplementary material for: What is an ideal mHealth for post-discharge home care of CHD: perspectives from primary caregivers, healthcare professionals, and project technical consultants: a qualitative research study
Source: Front Cardiovasc Med. 2026 Oct 20;13:1948803. doi: 10.3389/fcvm.2026.1948803 (PMC13727416; doi:10.3389/fcvm.2026.1948803)
Supplement: Supplementary file 1 [file Datasheet1.docx]

Consolidated criteria for reporting qualitative studies (COREQ): 32-item checklist

| **Item** | **No** | **Guide questions/description** | **Respond** |
| --- | --- | --- | --- |
| **Domain 1: Research team and reﬂexivity Personal Characteristics** | | | |
| Interviewer/facilitator | 1 | Which author/s conducted the interview or focus group? | The interviews were conducted by Yang Lan (YL). |
| Credentials | 2 | What were the researcher’s credentials? | A master's degree candidate in Nursing. |
| Occupation | 3 | What was their occupation at the time of the study? | Master's degree student |
| Gender | 4 | Was the researcher male or female? | male |
| Experience and training Relationship with participants | 5 | What experience or training did the researcher have? | Yang Lan had systematic training in qualitative methods. The Corresponding author (Ma，Doctoral degree ) served as a senior qualitative research scholar providing critical oversight. |
| Relationship established | 6 | Was a relationship established prior to study commencement? | No prior relationship. Participants were recruited via purposive sampling from the hospital wards. The principal investigator contacted them to introduce the study procedures. |
| Participant knowledge of the interviewer | 7 | What did the participants know about the researcher? | The principal investigator (YL) contacted potential participants to introduce the study procedures and research purpose. |
| Interviewer characteristics | 8 | What characteristics were reported about the interviewer/facilitator? | Yang Lan is a partial insider: involved in mHealth development alongside TC, which provided experiential familiarity but required ongoing reflexivity to avoid bias. |
| **Domain 2: study design** | | | |
| Methodological orientation and Theory  Participant selection | 9 | What methodological orientation was stated to underpin the study? | Reflexive Thematic Analysis (RTA) as developed by Braun and Clarke. |
| Sampling | 10 | How were participants selected? | Purposive sampling (see Table 1 for detailed inclusion/exclusion criteria). |
| Method of approach | 11 | How were participants approached? | The principal investigator (YL) contacted potential participants in person at the cardiology/cardiovascular surgery wards. A 24-hour consideration period was given. |
| Sample size | 12 | How many participants were in the study? | 16 participants in total: 7 Primary Caregivers (PC), 6 Healthcare Professionals (HP), and 3 Technical Consultants (TC). |
| Non-participation Setting | 13 | How many people refused to participate or dropped out? Reasons? | Refusal numbers were not formally tracked; no participants dropped out. |
| **Setting** | | | |
| Setting of data collection | 14 | Where was the data collected? | In a private room at a tertiary hospital in southwestern China. |
| Presence of non-participants | 15 | Was anyone else present besides the participants and researchers? | No, only the interviewer and the participant were present during the interviews. |
| Description of sample Data collection | 16 | What are the important characteristics of the sample? | PC: 6 mothers, 1 father (age 22-38, education primary to bachelor's). HP: 4 female nurses, 2 male physicians (age 22-47, titles junior to intermediate). TC: all male, aged 25-26, Master's in software engineering. Detailed demographics are in Appendix 2. |
| **Data collection** | | | |
| Interview guide | 17 | Were questions, prompts, guides provided by the authors? Was it pilot tested? | Yes. Separate semi-structured interview guides were developed for each group, pilot-tested (3 pilot interviews), and revised. Final guides are provided in Supplementary Appendix 1. |
| Repeat interviews | 18 | Were repeat interviews carried out? If yes, how many? | No. Only single one-on-one in-depth interviews were conducted. |
| Audio/visual recording | 19 | Did the research use audio or visual recording to collect the data? | Yes. All interviews were audio-recorded with participant consent. |
| Field notes | 20 | Were ﬁeld notes made during and/or after the interview or focus group? | No formal field notes; audio recordings and transcriptions were used instead. |
| Duration | 21 | What was the duration of the interviews or focus group? | Lasted 20 to 45 minutes. |
| Data saturation | 22 | Was data saturation discussed? | Yes, but reframed. We explicitly state that "saturation" is ill-fitting for Reflexive Thematic Analysis; instead, we justify sample size using the concept of "data adequacy". |
| Transcripts returned | 23 | Were transcripts returned to participants for comment and/or correction? | No. To minimise participant burden, transcripts were not returned for verification. |
| **Domain 3: analysis and ﬁndingsz** | | | |
| **Data analysis** | | | |
| Number of data coders | 24 | How many data coders coded the data? | Two human researchers (Yang Lan performed full analysis; Ma provided critical oversight) + Two Large Language Models (Qwen-Max and DeepSeek R1) used as supplementary brainstorming assistants. |
| Description of the coding tree | 25 | Did authors provide a description of the coding tree? | Yes. Themes are described in the Results section, and the cross-checking process among human and AI is visually summarized in Figure 1. |
| Derivation of themes | 26 | Were themes identiﬁed in advance or derived from the data? | Inductive (data-driven). Themes were generated iteratively from line-by-line coding of the transcripts, combined with team consensus discussions. |
| Software | 27 | What software, if applicable, was used to manage the data? | NVivo 12 was used to manage the qualitative data. |
| Participant checking | 28 | Did participants provide feedback on the ﬁndings? | No. In line with the reflexive thematic analysis approach, member checking was not performed. |
| **Reporting** | | | |
| Quotations presented | 29 | Were participant quotations presented to illustrate the themes / ﬁndings? Was each quotation identiﬁed? e.g. participant number | Yes. Verbatim quotations (e.g., PC 1, HP 3, TC 1) are presented in the Results section for each theme. |
| Data and ﬁndings consistent | 30 | Was there consistency between the data presented and the ﬁndings? | Yes. Each theme is clearly supported by representative quotes from all three stakeholder groups. |
| Clarity of major themes | 31 | Were major themes clearly presented in the ﬁndings? | Yes. Six overarching themes are clearly named and defined: (1) Tailored to individual; (2) Easy to use; (3) Media in convergence; (4) At the ready; (5) One clear voice; (6) Emotional support on demand. |
| Clarity of minor themes | 32 | Is there a description of diverse cases or discussion of minor themes? | No. |
