# Supplementary material for: What is an ideal mHealth for post-discharge home care of CHD: perspectives from primary caregivers, healthcare professionals, and project technical consultants: a qualitative research study
Source: Front Cardiovasc Med. 2026 Oct 20;13:1948803. doi: 10.3389/fcvm.2026.1948803 (PMC13727416; doi:10.3389/fcvm.2026.1948803)
Supplement: Supplementary file 3 [file Table2.docx]

**Appendix 1. Interview guide**

Part1

Primary Caregivers

- What were the greatest difficulties you encountered from the child’s diagnosis to surgery? What approaches did you generally adopt to address these difficulties?
- What do you perceive as the respective advantages of directly consulting physicians versus using mobile healthcare services?
- Have you ever used mobile‑health‑related applications to search for health‑related questions? Could you share your user experience? What was your most prominent impression during this process?

- What emotional impacts does the use of mobile healthcare exert on you? Are these impacts predominantly positive or negative?

- What difficulties or confusing issues have you encountered while using mobile healthcare services? How did you deal with them?

- Among the mobile healthcare products you have come into contact with, what aspects do you like most? What are these aspects specifically? Are there any functions that motivate your continuous use of such products?

- What functions of mobile healthcare do you most frequently utilize?

- Do you have doubts about the information provided by mobile healthcare services? What would you do when you question its authenticity?

- What functions do you believe an ideal mobile‑health product should possess?

- In terms of the mobile‑health products you have used, what aspects do you think could be improved?

Healthcare Professionals

- Have you come‑across mHealth‑related tools or software in your professional work? Please briefly share your practical experience in using them

- Do you perceive mHealth tools as a help or a burden to your work? In what main aspects are these effects reflected?

- Have you encountered any problems or frustrations while using mHealth tools?

- Do you believe that the content provided by current mHealth tools can meet patients’ needs?

- Do you believe mHealth tools can play a role in psychological counselling or emotional support?

- What functions do you believe a good mHealth tool should possess?

- In terms of the mobile‑health products you have used, what aspects do you think could be improved?

Part 2

Part 3

Technical Consultants

- What are your most significant perceptions when using mHealth products?

- What do you perceive as the greatest challenges in the development of mHealth products?

- What do you perceive as the greatest strengths of mHealth products?

- What do you perceive as the main limitations of current mHealth products?

- What do you perceive as the reasons why some well‑designed mHealth applications have low user engagement?

- What do you perceive as the differences between user‑experience (UX) design in healthcare contexts and that of other applications?

- What do you perceive as the means by which empathy and psychological support can be delivered within mHealth products?
