# Supplementary material for: What is an ideal mHealth for post-discharge home care of CHD: perspectives from primary caregivers, healthcare professionals, and project technical consultants: a qualitative research study
Source: Front Cardiovasc Med. 2026 Oct 20;13:1948803. doi: 10.3389/fcvm.2026.1948803 (PMC13727416; doi:10.3389/fcvm.2026.1948803)
Supplement: Supplementary file 4 [file Table3.docx]

**Appendix 2. Demographic Characteristics of Participating**

Part1 Demographic Characteristics of Primary Caregivers

| ID | Sex | Age | Relationship with the child | Educational level | Residential location of participant | Diagnosis of child |
| --- | --- | --- | --- | --- | --- | --- |
| PC1 | Female | 23 | Mother-daughter | Associate degree | Rural | Ventricular septal defect |
| PC2 | Female | 38 | Mother-daughte | Primary school diploma | Urban | Atrial septal defect+ ventricular septal defect |
| PC3 | Female | 22 | Mother-daughte | High school diploma | Urban | Atrial septal defect |
| PC4 | Female | 24 | Mother-daughte | Bachelor’s degree | Urban | Bicuspid aortic valve |
| PC5 | male | 27 | Father-daughte | Bachelor’s degree | Rural | Ventricular septal defect |
| PC6 | Female | 22 | Mother-daughte | High school diploma | Rural | Ventricular septal defect |
| PC7 | Female | 25 | Mother-daughte | Bachelor’s degree | Rural | Atrial septal defect |

Part2 Demographic Characteristics of Healthcare Professionals

| ID | Sex | Age | Job Category of HP | Educational level | Professional Title of HP | Specialty Working Years of HP |
| --- | --- | --- | --- | --- | --- | --- |
| HP1 | Female | 22 | Nurse | Bachelor’s degree | Junior nurse | 2 |
| HP2 | Female | 22 | Nurse | Bachelor’s degree | Junior nurse | 2 |
| HP3 | Female | 35 | Nurse | Bachelor’s degree | Supervisor nurse | 10 |
| HP4 | Female | 31 | Nurse | Bachelor’s degree | Supervisor nurse | 7 |
| HP5 | male | 47 | Doctor | Bachelor’s degree | Attending physician | 17 |
| HP5 | male | 31 | Doctor | Master’s degree | Attending physician | 4 |

Part3 Demographic Characteristics of Technical Consultants

| ID | Sex | Age | Academic Major | Educational level | Role Assumed in Previous mHealth Projects |
| --- | --- | --- | --- | --- | --- |
| TC1 | male | 25 | Software Engineering | Master’s degree | Back-End Development Engineer |
| TC2 | male | 24 | Software Engineering | Master’s degree | Requirements Analyst |
| TC3 | male | 27 | Software Engineering | Master’s degree | Back-End Development Engineer |
